# Supplementary material for: Evaluating healthcare professionals’ readiness for community-based kangaroo mother care in resource-limited setting
Source: PLoS One. 2026 May 11;21(5):e0349104. doi: 10.1371/journal.pone.0349104 (PMC13160445; doi:10.1371/journal.pone.0349104)
Supplement: S1 File — (DOCX) [file pone.0349104.s001.docx]

Inclusivity in global research

PLOS’ policy on inclusivity in global research aims to improve transparency in the reporting of research performed outside of researchers’ own country or community and ensures that PLOS publications reporting global research adhere to high standards for research ethics and authorship. Authors of relevant research articles may be asked to complete the questionnaire below, which outlines ethical, cultural, and scientific considerations specific to inclusivity in global research. This questionnaire may be requested when researchers have travelled to a different country to conduct research, if research uses samples collected in another country, research with Indigenous populations or their lands, or if research is on cultural artefacts. Researchers travelling to another country solely to use laboratory equipment will not normally be required to complete the questionnaire. However, the questionnaire can be requested at the journal’s discretion for any submission – if you have been requested to complete this questionnaire by the PLOS journal you submitted to, please do so.

Please complete the questionnaire below and include this as a Supporting Information file with your manuscript. Note that if your paper is accepted for publication, this checklist will be published with your article in the supporting information files. Please ensure that you reference the checklist in the main body of your manuscript. We suggest adding a subsection ‘Inclusivity in global research’ to your Methods section and adding the following sentence: “Additional information regarding the ethical, cultural, and scientific considerations specific to inclusivity in global research is included in the Supporting Information (SX Checklist)”

The questions have been designed to be applicable to a wide range of study types, and there are subsections for both human subjects research and non-human subjects research. If any of the questions are not relevant to your research please mark them as “N/A” as appropriate.

**Ethical considerations, permits and authorship**

*This section is applicable to all research types.*

Provide details as to who granted permissions and/or consent for the study to take place in the Methods section of your manuscript. This should include the names of **all** ethics boards, governmental organizations, community leaders or other bodies that provided approval for the study. If individuals provided approval refer to these people by their role or title but do not list their name(s).

Reported on page number: Page 12, lines 236- 246

If there were any deviations from the study protocol after approval was obtained please provide details of these changes in the Methods section of your manuscript.
Did this study involve local collaborators that are residents of the country where the research was conducted or members of the community studied? If you do not have any authors from said communities, please provide an explanation for this below.

Reported on page number: N/A

Yes, the primary author resides in the country and is a member of the studied community.

Everyone listed as an author should meet PLOS’ criteria for authorship and all individuals who meet these criteria should be included in the author byline, rather than the acknowledgements. For further information please see the journal’s Authorship Policy.

**Human subjects research (e.g. health research, medical research, cross-cultural psychology)**

Did you obtain written informed consent from a representative of the local community or region before the research took place? How did you establish who speaks for the community? Details of written informed consent obtained from study participants should be reported separately in the Methods section of your manuscript.

The study secured a written permission letter from the health research institutes of the Tigray region. This letter was distributed to subdistricts and subsequently directed to each health facility and community. Data collection was conducted online using Qualtrics, with assistance from research assistants. Two research assistants were recruited locally to facilitate this process. Participants were informed that completing the online data collection would constitute informed consent, a process approved by both the local Aksum University Health Research Ethics Committee and the Griffith University Human Research Ethics Committee.

How did members of the local community provide input on the aims of the research investigation, its methodology, and its anticipated outcome(s)?

As previously mentioned, the first author resides in the community being studied and played a pivotal role in shaping the research objectives and developing the online survey methodology. This team member perspective significantly enhanced the survey's cultural relevance and contextual appropriateness, influencing aspects such as item wording, response formats, and the mode of online delivery. As a result, the anticipated outcomes of the study were strengthened by ensuring that the data collection process was in alignment with local practices and realities. Additionally, locally based research assistants supported the online survey by facilitating participant access, clarifying survey items when necessary, and promoting culturally sensitive engagement with the digital platform.

When engaging with the local community, how did you ensure that the informed consent documents and other materials could be understood by local stakeholders?

In our engagement with the local community, we implemented several measures to ensure that informed consent documents and study materials were accessible and comprehensible to local stakeholders. All participant information sheets and consent forms were translated into Tigrigna and subsequently back-translated into English to guarantee both conceptual and linguistic accuracy. We utilised plain, non-technical language and clarified key concepts using terms familiar to the community. Before data collection commenced, the materials were reviewed by local healthcare professionals and community members to assess their clarity and cultural appropriateness. For the online survey, consent information was presented in a clear, step-by-step format at the outset, and participants were encouraged to seek clarification from locally based team members and research assistants whenever necessary.

Will the findings of the research be made available in an understandable format to stakeholders in the community where the study was conducted (e.g. via a presentation, summary report, copies of publications, etc.)? Please provide details of how this will be achieved.

Initially, a summary of the key findings and practical implications will be prepared in both Tigrigna and English, and shared with local health facilities, health extension workers, and relevant community leaders. Additionally, summary reports and copies of published articles will be provided to local health offices and participating facilities. Where feasible, electronic copies will be distributed via commonly used communication platforms to facilitate access. Furthermore, we plan to present our findings at both local and international conferences to enhance dissemination. Through these various dissemination strategies, the study aims to ensure that the findings are clear, locally relevant, and valuable for informing community-based kangaroo mother care practices and decision-making.

**Non-human subjects research using specimens/ animals collected as part of the study, or those housed in archival collections. Examples include archaeology, paleontology, botany and zoology.**

Did the permission you obtained from a local authority to perform the study include an agreement on access to outputs and benefit sharing? This may include procedures to enable fair distribution of the benefits and resources arising from the research performed. Please include any details of Prior Informed Consent and Benefit Sharing Agreements obtained. These may be required by field-specific regulations, for example the Convention on Biological Diversity (CBD) and the associated Nagoya Protocol.

N/A

If the material used in your study was imported, please A) provide the year it was imported and B) indicate whether permits were obtained to import/export the materials used, C) provide details of any permits obtained. If this information is not available, please indicate this.

N/A

If you used archival specimens, please state how the material used in your study was acquired by the institute it is held in and provide details of any permits obtained for the original excavations/ sample collection. If this information is not available, please indicate this.

N/A

How was the potential cultural significance of the materials collected in your study to local communities considered in your research design? Were Indigenous peoples and/or local researchers and institutions involved with archaeological excavations / collection of specimens? If so, please provide a description of their involvement.

N/A

If your manuscript includes photographs of human remains please indicate whether authors obtained permission from descendants or affiliated cultural communities to do so.

N/A
