# Supplementary material for: Evaluating healthcare professionals’ readiness for community-based kangaroo mother care in resource-limited setting
Source: PLoS One. 2026 May 11;21(5):e0349104. doi: 10.1371/journal.pone.0349104 (PMC13160445; doi:10.1371/journal.pone.0349104)
Supplement: S3 File — (DOCX) [file pone.0349104.s003.docx]

Thank you for participating in this study. This study will help us understand health workers’ knowledge, perceptions, and practices related to Community-based Kangaroo Mother Care (cKMC).

You may already be familiar with Kangaroo Mother Care (KMC), which is a method of caring for premature or low birthweight (LBW) babies that involves continuous skin-to-skin contact for at least 8 hours per day and on-demand exclusive breastfeeding.

This study is about community-based Kangaroo Mother Care (cKMC), which refers to applying KMC principles in the home or community settings for babies who are either born at home or have been discharged from health facilities.

This questionnaire is divided into two sections:

1. Questions about you: This section will ask about your background to help us understand the context of your responses.

2. Statements about cKMC: This section includes statements to assess your knowledge, perceptions, and practices related to cKMC. You will be asked to indicate how much you agree with each statement using a 5-point scale.

For full details about this study, including ethics approval, please [click this link](https://griffithuw.qualtrics.com/ControlPanel/File.php?F=F_4hBY3wW0xM03Owc) to access the participant information form and research team contact details.

Your responses are valuable to this research and are completely anonymous. If you have any questions, please feel free to contact our research team.

Thank you for your time and participation!

| Question | | Response | | | | Go to | |
| --- | --- | --- | --- | --- | --- | --- | --- |
| Q1. Age in years | | _____________ | | | |  | |
| Q2. Residence | | 1. Urban 2. Rural | | | |  | |
| Q3. Profession | | 1. Nurse 2. Midwife 3. Health extension 4. Health officer 5. Medical Doctor | | | |  | |
| Q4. Highest Qualification | | 1. Certificate 2. Diploma 3. Bachelor 4. Master 5. Doctorate | | | |  | |
| Q5. How long have you been working in your profession? | | ____________________ | | | |  | |
| Q6. Prior to completing the questionnaire, have you heard of the Kangaroo Mother Care practice? | | 1. Yes 2. No | | | |  | |
| Q7. Prior to completing the questionnaire, have you heard of the Community-based Kangaroo Mother Care practice? | | 1. Yes 2. No | | | |  | |
| Q8. Have you had experience supporting families in Community-based Kangaroo Mother Care? | | 1. Yes 2. No | | | |  | |
| Q9. Have you seen the “Kangaroo Mother Care Technical and Implementation Guideline” released in January 2023 by the Ministry of Health of Ethiopia? | | 1. Yes 2. No………………………… | | | | ‘No’ skip to question 11 | |
| Q10. If you have seen the “Kangaroo Mother Care Technical and Implementation Guideline”, how did you obtain it? **(Select all that apply)** | | 1. Email 2. Telegram 3. WhatsApp 4. Training 5. Colleague 6. Employer or in the workplace 7. Non-governmental organisation 8. If other, please specify._______________ | | | |  | |
| Q11. Have you had experience with KMC in a health facility setting? | | 1. Yes 2. No | | | |  | |
| Q12. Have you received training about cKMC? | | 1. Never 2. One time 3. Two and more | | | |  | |
| Q13. Do you visit families at home to support them in caring for preterm and low birth weight babies? | | 1. Yes 2. No | | | |  | |
| Q14. Do you use cKMC to transport preterm and low birth weight neonates? | | 1. Yes 2. No………………………………. | | | | ‘No’ skip to question 16 | |
| Q15. If yes question 14, when do you use Community-based Kangaroo Mother Care (cKMC) to transport preterm and low birth weight babies? **(Select all that apply)** | | 1. From home to places in the community (eg. church, market, shopping, social gatherings) 2. From home to health facilities 3. From health facility to another health facility 4. From health facility to home 5. All of the above | | | |  | |
| Q16. Have you ever helped raise awareness about cKMC in the community? | | 1. Yes 2. No…………………………………. | | | | ‘No’ skip to question 18 | |
| Q17 If yes question 16, which platforms have you used to create awareness about cKMC? **(Select all that apply)** | | 1. Antenatal care (ANC) Visit 2. Pregnant women conference 3. Village health leaders 4. Health development army 5. If other specify _____________________ | | | |  | |
| Please consider the following statements about Community-based Kangaroo Mother Care (cKMC) carefully, from Strongly Disagree to Strongly Agree, and indicate how much you agree with each statement. | | | | | | | |
|  | 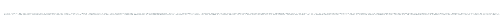Strongly Disagree | | Disagree | Agree | Strongly agree | | Un sure/ uncertain/ not know |
| Q18. I am aware of the KMC practice, which comprises skin-to-skin contact and exclusive breastfeeding for premature and/or low birth weight neonates. | 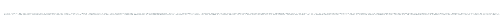 | |  |  |  | |  |
| Q19. cKMC helps premature and low birth weight babies breathe better. |  | |  |  |  | |  |
| Q20. cKMC is recommended for a minimum of eight hours per day to promote the health of premature and /or low birth weight neonates. |  | |  |  |  | |  |
| Q21. cKMC helps regulate the heartbeat of premature and/or low birth weight babies. |  | |  |  |  | |  |
| Q22. Neonates may experience slight temperature fluctuation during cKMC |  | |  |  |  | |  |
| Q23. I am aware of specially designed baby wraps that help parents provide continuous skin-to-skin care safely for their newborns. |  | |  |  |  | |  |
| Q24. cKMC is a safe and effective method for caring for premature and low birth weight neonates. |  | |  |  |  | |  |
| Q25. cKMC helps mothers successfully breastfeed premature and low birth-weight neonates. |  | |  |  |  | |  |
| Q26. KMC can be practised at home OR in the community for premature and low birth weight neonates. |  | |  |  |  | |  |
| Q27. cKMC can help increase a mother’s milk supply for her newborn. |  | |  |  |  | |  |
|  | Strongly Disagree | | Disagree | Agree | Strongly agree | | Un sure/ uncertain/ not know |
| Q28. I have received sufficient training in cKMC practice. |  | |  |  |  | |  |
| Q29. I provide information and promote cKMC to families in the community. |  | |  |  |  | |  |
| Q30. I actively encourage and support mothers in practising cKMC as part of my healthcare responsibilities. |  | |  |  |  | |  |
| Q31. I encourage fathers to practice cKMC. |  | |  |  |  | |  |
| Q32. I feel competent in assisting and supporting families with cKMC practice |  | |  |  |  | |  |
| Q33. Assisting and supporting families with cKMC practice is part of my healthcare responsibilities. |  | |  |  |  | |  |
| Q34. My workplace provides the necessary resources and support to assist families with cKMC. |  | |  |  |  | |  |
| Q35. I am committed to promoting cKMC so that families can understand and appreciate its important benefits. |  | |  |  |  | |  |
| Q36. I feel confident talking to families about cKMC when they have concerns about this practice. |  | |  |  |  | |  |
|  | 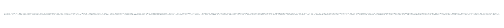Strongly Disagree | | Disagree | Agree | Strongly agree | | Un sure/ uncertain/ not know |
| Q37. I believe there are specific medical conditions where cKMC may not be suitable. |  | |  |  |  | |  |
| Q38. Traditional carrying babies on the back is a barrier to cKMC |  | |  |  |  | |  |
| Q39. With guidance and encouragement, families are willing to adopt cKMC in the community. |  | |  |  |  | |  |
| Q40. Families who practice cKMC feel supported and accepted within their community. |  | |  |  |  | |  |
| Q41. Mothers feel comfortable practising cKMC in appropriate settings that ensure their privacy and modesty. |  | |  |  |  | |  |
| Q42. Families feel confident handling their baby safely while practising cKMC with the proper guidance. |  | |  |  |  | |  |
| Q43. cKMC can be integrated into family caregiving routines without disrupting daily tasks and responsibilities. |  | |  |  |  | |  |
| Q44. Fathers can support mothers in practising cKMC while balancing household responsibilities and childcare. |  | |  |  |  | |  |
| Q45. Others family members can support mothers in practising cKMC while balancing household responsibilities and childcare. |  | |  |  |  | |  |
| Q46. Families perceive cKMC to be time-consuming. |  | |  |  |  | |  |
| Q47. With appropriate resources and support, cKMC can be effectively practised in remote areas. |  | |  |  |  | |  |
| Q48. Strong collaboration between health facilities and the community can enhance cKMC implementation. |  | |  |  |  | |  |
| Q49. Increased financial support from governments and non-governmental organisations can further strengthen cKMC training for healthcare providers. |  | |  |  |  | |  |
| Q50. Time constraints can impact the ability of health professionals to support families practising cKMC. |  | |  |  |  | |  |
