# Supplementary material for: Evaluating healthcare professionals’ readiness for community-based kangaroo mother care in resource-limited setting
Source: PLoS One. 2026 May 11;21(5):e0349104. doi: 10.1371/journal.pone.0349104 (PMC13160445; doi:10.1371/journal.pone.0349104)
Supplement: Attachment — Submitted filename: Response to reviewers and editor 28012026.docx [file pone.0349104.s006.docx]

| **Response to reviewers and editors** | |
| --- | --- |
| **PONE-D-25-57353** | |
| **Evaluating healthcare professionals’ readiness for community-based kangaroo mother care in resource-limited setting** | |
| **Reviewer or editor comments** | **Response** |
| Please ensure that your manuscript meets PLOS ONE's style requirements, including those for file naming. The PLOS ONE style templates can be found at  <https://journals.plos.org/plosone/s/file?id=wjVg/PLOSOne_formatting_sample_main_body.pdf> and  [https://journals.plos.org/plosone/s/file?id=ba62/PLOSOne_formatting_sample_title_authors_affiliations.pdf](https://track.editorialmanager.com/CL0/https:%2F%2Fjournals.plos.org%2Fplosone%2Fs%2Ffile%3Fid=ba62%2FPLOSOne_formatting_sample_title_authors_affiliations.pdf/1/010f019b81fc523a-75abe82f-6972-4c3d-8c8f-89d1bd271d75-000000/2LRPpraQtAfgi-Mq4-sf6rsbE2gF3XyxfRUP-8dCb3E=244) | We revised the font size of headings and subheadings throughout the document, including the abstract, introduction, materials and methods, results, discussion, conclusion, references, and acknowledgment. |
| Please include a complete copy of PLOS’ questionnaire on inclusivity in global research in your revised manuscript. Our policy for research in this area aims to improve transparency in the reporting of research performed outside of the researchers’ own country or community. The policy applies to researchers who have travelled to a different country to conduct research, research with Indigenous populations or their lands, and research on cultural artefacts. The questionnaire can also be requested at the journal’s discretion for any other submissions, even if these conditions are not met. Please find more information on the policy and a link to download a blank copy of the questionnaire here: <https://journals.plos.org/plosone/s/best-practices-in-research-reporting>. Please upload a completed version of your questionnaire as Supporting Information when you resubmit your manuscript. | The PLOS’ questionnaire addressing inclusivity in global research has been submitted as a supplementary document and subsequently cited within the manuscript. See lines 232-234  A version of the questionnaire is included as a supplementary file named HiPECK-cKMC Instrument. |
| We note that the grant information you provided in the ‘Funding Information’ and ‘Financial Disclosure’ sections does not match. When you resubmit, please ensure that you provide the correct grant numbers for the awards you received for your study in the ‘Funding Information’ section. | This study did not receive specific grant funding. We have now ensured that the financial disclosure and funding information are aligned. |
| We note that your Data Availability Statement is currently as follows: All relevant data are within the manuscript and its Supporting Information files.  Please confirm at this time whether or not your submission contains all raw data required to replicate the results of your study. Authors must share the “minimal data set” for their submission. PLOS defines the minimal data set to consist of the data required to replicate all study findings reported in the article, as well as related metadata and methods (<https://journals.plos.org/plosone/s/data-availability#loc-minimal-data-set-definition>). For example, authors should submit the following data: - The values behind the means, standard deviations and other measures reported; - The values used to build graphs; - The points extracted from images for analysis. Authors do not need to submit their entire data set if only a portion of the data was used in the reported study.  If your submission does not contain these data, please either upload them as Supporting Information files or deposit them to a stable, public repository and provide us with the relevant URLs, DOIs, or accession numbers. For a list of recommended repositories, please see <https://journals.plos.org/plosone/s/recommended-repositories>. If there are ethical or legal restrictions on sharing a de-identified data set, please explain them in detail (e.g., data contain potentially sensitive information, data are owned by a third-party organization, etc.) and who has imposed them (e.g., an ethics committee). Please also provide contact information for a data access committee, ethics committee, or other institutional body to which data requests may be sent. If data are owned by a third party, please indicate how others may request data access. | We have submitted the dataset used for the main analysis as a supplementary file.  The dataset submitted contained all raw data that would be required to replicate the results of the study. The name of file was EFA Dataset. |
| Please revise your manuscript to clearly and explicitly present your study objectives and research questions. Clearly stating these elements will help readers understand the purpose and scope of your research. Additionally, we encourage you to carefully review the presentation of your results to ensure clarity, logical flow, and alignment with your objectives. Clear tables, figures, and narrative descriptions can significantly enhance the readability and impact of your findings. | Thank you for your insightful suggestions. We have revised the manuscript to clearly state the objective: to enhance the understanding of the purpose and scope of our research. “This study primarily aimed to assess the readiness of healthcare professionals for the implementation of cKMC. In addition, this study developed and performed preliminary validation of an instrument to measure healthcare professionals’ capability, perception, knowledge, and engagement with cKMC.” on lines 25-28 in the abstract and lines 97-101 in the introduction, |
| Reviewer 1 |  |
| 1. Please explain the term “certificate” in the context of the qualification | The following text has been added to the manuscript on lines 267 and 268: “Certificate is the entry-level qualification for rural health extension workers. It requires completion of 10th grade, one year of formal pre-service training, and passing the Level III Certificate of Competency test (COC).” |
| 2. The Results section can be summarised more succinctly. Much of its content duplicates what is already reported in the tables. | We addressed this by summarising the pertinent results more succinctly, explaining and describing them. Some examples we deleted from the manuscript “The highest within this factor was for the item ‘I actively encourage and support mothers in practising cKMC as part of my healthcare responsibilities’ 3.86 (0.95).” and the description part of the tables was minimised to reflect the main results. |
| 3. When reporting attainable scores, provide both the minimum and maximum scores. Reporting only the maximum can be misleading; for example, in line 298, 45 should be reported as 9–45 | We made these suggested corrections on lines 309, 319, and 339. |
| 4. Table 1 • Please include the number of participants at the end of the table title. | We included the number of participants (593) in the title of Table 1. |
| 5. For dichotomous variables, reporting one category is sufficient and will shorten the table. | We believed that providing a single response for dichotomous questions could mislead readers, as it is difficult to determine if they are in a dichotomous situation; therefore, we chose to leave it unchanged. |
| 6. Consider abbreviating and shortening the names of variables. | We shortened some variable names. See Table 1. |
| 7. Some items, including item 2, may not be suited for the reported domain (capability). Item 9 does not appear to fit with the response format. | Thank you for your suggestions. Item 2 reflects professional role clarity and perceived ability to assist and support families in practising cKMC, indicating professional capability to support and assist. Regarding Item 9, it is modified. “I have received sufficient training in cKMC practice.” All the items were reviewed by seven panel members with strong experience in development and psychometric tool evaluation. |
| 8. Please explain how each item was scored and indicate which items are reverse-scored. State the attainable (possible) score for each domain. | We explained how each item was scored in the Method section, lines 159-164 “A Likert scale was chosen for its effectiveness in capturing behaviours, attitudes, and perceptions. Each item was presented as a declarative statement with five response options: Strongly Disagree (1), Disagree (2), Unsure/Uncertain (3), Agree (4), and Strongly Agree (5). The neutral midpoint (‘Neither agree nor disagree’) was intentionally excluded to discourage indirect responses and encourage directional responses, while the ‘Unsure/Uncertain’ option was retained to differentiate non-attitudes or lack of knowledge from genuine neutrality [27].”  We assigned an identification code to items used in reverse scoring in Table 3, marked with asterisks (*). The attainable (possible) score was documented in the description of results on lines 309, 319, 327, and 339, and we have also included it in Table 3. |
| 9. You state that a Kruskal–Wallis test was used for between-group comparisons due to skewness. In Table 3, however, you report the mean (SD). When skewness is present, mean (SD) is not the best descriptive summary; consider reporting the median with interquartile range (Q1–Q3) instead. | As distributions were skewed and non-parametric tests were used, we have revised Table 3 to report medians and interquartile ranges for all continuous variables. |
| 10. Table 4 • Descriptive results should also be reported. For example, although the table shows a significant difference between participants from urban and rural areas in terms of capability score, it does not indicate which group has the higher score. | We have revised Table 4 to include descriptive statistics (median and interquartile range) for both urban and rural groups and for variables, which now clearly indicate which group has the higher capability score. |
| 11. Table 5 • The reference group should be specified for each predictor. | We specified the reference group in Table 5. |
| 12. Report ceiling effects where relevant. | We added ceiling effects for each item. We calculated the maximum score percentage from all study participants.  Ceiling effect = the number of rated maximum scores x 100 . the total number of participants  See Table 3. |
| 13. Stability is also recommended as an indicator of reliability. If you have not assessed it, acknowledge this in the limitations subsection. | Thank you for your feedback. We did not assess stability using a test–retest approach; this has now been acknowledged as a limitation, with a recommendation for future research to examine item stability over time. See line 573 |
| Reviewer 2 |  |
| 1. The manuscript would benefit from a brief clarification on why PCA rather than common factor analysis was selected, and how this choice may affect interpretation. | We have clarified our rationale for selecting PCA rather than common factor analysis, as the aim was data reduction into underlying dimensions rather than theory testing or hypothesis confirmation. We also note that PCA components represent weighted combinations of observed variables rather than latent constructs, and this has been clarified in the Methods. See lines 191 and 195. |
| 2. Some items in the Engagement subscale show lower internal consistency; a short discussion on plans for future refinement or item expansion would strengthen the paper. | We have added the following text to the limitations section: “Engagement has emerged as a significant factor; however, the subscale consists of only three items and recorded the lowest Cronbach's Alpha value of 0.67. This may not adequately capture the intricacies of systemic enablers and barriers. As a result, there is a need for expansion to encompass aspects such as leadership, policy integration, and resource allocation.” Lines 564-572. |
| 3. Minor grammatical and stylistic edits are needed throughout the manuscript to improve readability. | We revised the document to address your suggestion on minor grammatical and stylistic edits. |
| 4. The discussion could be strengthened by briefly addressing how the tool may be adapted or validated in other low-resource or non-Ethiopian contexts. | We made modifications to the discussion based on this feedback. The following text was added “While the instrument was developed for the Ethiopian context, the tool has potential relevance for other low-resource settings; however, cultural adaptation and context-specific validation are required before wider application.” See lines 551 -556 |
| Reviewer 3 |  |
| 1. The manuscript needs to clearly state whether the primary objective is to develop and preliminarily validate an instrument or to evaluate healthcare professionals’ readiness. The authors should clearly distinguish between instrument development as the primary objective and exploratory description of readiness as a secondary output. Readiness-related findings should be framed cautiously and not as confirmatory conclusions. | Thank you for your valuable feedback. We modified the primary objective. See lines 25-28 in the abstract and lines 97-101 in the introduction “This study primarily aimed to assess the readiness of healthcare professionals for the implementation of cKMC. In addition, this study developed and performed preliminary validation of an instrument to measure healthcare professionals’ capability, perception, knowledge, and engagement with cKMC.” |
| 2. The manuscript indicates that 14 domains from the Theoretical Domains Framework were grouped into four constructs (capability, perception, knowledge, engagement). The rationale for this reduction is insufficiently explained. A clear explanation or a mapping table linking the original domains to the final constructs would improve conceptual clarity. | We explained the conceptual linkage between the 14 Theoretical Domains Framework (TDF) domains and the four final constructs.  In this study, the TDF domains were used exclusively to guide item development, ensuring comprehensive theoretical coverage of behavioural determinants relevant to community-based kangaroo mother care. The TDF domains were not assumed to represent the final measurement structure. Following data collection, principal component analysis (PCA) was conducted to identify empirically coherent groupings of items based on shared variance. The number of components was determined using parallel analysis, which supported a four-component solution. These components represent empirically derived higher-order constructs, each comprising items originating from multiple TDF domains.  Construct labels were assigned based on the dominant conceptual content of the items loading onto each component. Specifically, components were interpreted according to whether their items primarily reflected knowledge acquisition, perceived ability to perform cKMC, evaluative beliefs about cKMC, or active involvement in its implementation. |
| 3. Interpretation of Statistical Findings   Several associations reach statistical significance but are accompanied by very small effect sizes, indicating limited practical relevance. The discussion should therefore differentiate more clearly between statistical significance and real-world importance. | Thank you for your suggestion. We acknowledge that some of the statistically significant findings were associated with negligible effect sizes. To further validate these results, we conducted a generalised linear model analysis, which revealed that most outcomes were no longer statistically significant, as illustrated in Table 5. We approached our discussion section with care to accurately represent both statistical significance and real-world relevance. |
| 4. Terminology related to cKMC is used inconsistently (e.g., “community-based KMC,” “cKMC,” “home-based KMC”). Consistent use of a single term throughout the manuscript would improve clarity. | We revised the manuscript to use cKMC exclusively for consistency. |
| 5. Consent process should be stated earlier in the Methods   Example wording: Information regarding informed consent (e.g., that questionnaire completion implied consent) is mentioned later in the manuscript. For clarity, this information should be stated earlier in the Methods section. | Thank you for your feedback. The consent statement is now presented in the Methods section, under the Ethical Considerations subheading. Appears “Participants understood that completing the online survey questionnaire constituted consent.” See lines 245 and 246. |
