# Supplementary material for: Evaluating healthcare professionals’ readiness for community-based kangaroo mother care in resource-limited setting
Source: PLoS One. 2026 May 11;21(5):e0349104. doi: 10.1371/journal.pone.0349104 (PMC13160445; doi:10.1371/journal.pone.0349104)
Supplement: Attachment — Submitted filename: CKMC manuscript.docx [file pone.0349104.s005.docx]

CKMC manuscript

Thank you for the opportunity to review this overall well-organized and written manuscript. KMC clearly defined and the rationale for community and home-based implementation is significant in a LMIC such as Ethiopia.

Unclear which HCP would be involved in implementation of KMC in community setting? Although HEWs are mentioned in methods section their role is unclear to many readers, unclear what capacity they would serve.

In the methods and sampling process, it seems the strata’s could be potentially biased. How was this addressed?

The focus group methodology needs more detail such as who led the session, whether trained in this methodology and example of participants included per session.

Provide the rationale for including which prior KMC surveys were utilized and how the deductive process of not including items were conducted from these surveys.

Statistical approaches appear to be appropriate for aims of paper.

The ethical considerations need to be moved before data collection, perhaps at end of sample.

The discussion and implications were well written with excellent policy recommendations moving forward.
