# Supplementary material for: Evaluating healthcare professionals’ readiness for community-based kangaroo mother care in resource-limited setting
Source: PLoS One. 2026 May 11;21(5):e0349104. doi: 10.1371/journal.pone.0349104 (PMC13160445; doi:10.1371/journal.pone.0349104)
Supplement: Attachment — Submitted filename: Response to Reviewer PLOS ONE 230426.docx [file pone.0349104.s007.docx]

**Response to Reviewer**

**Evaluating healthcare professionals’ readiness for community-based kangaroo mother care in resource-limited setting**

**[PONE-D-25-57353R1] - [EMID:133aa923351b60a4]**

| Reviewer 3 Feedback | Responses |
| --- | --- |
| Unclear which HCP would be involved in the implementation of KMC in the community setting? Although HEWs are mentioned in the methods section, their role is unclear to many readers, unclear in what capacity they would serve. | Thank you for this important comment. In community settings, KMC implementation primarily involves health extension workers (HEWs), with support from midwives, nurses, health officers, and physicians working in primary health care.  HEWs play a central role as they are certificate trained health professionals embedded within the community through the national health extension programme. Their responsibilities include home‑to‑home service delivery, maternal and newborn health education, postnatal follow‑up, identification of premature and low‑birth‑weight infants, counselling on newborn care, and linkage between households and health facilities. Midwives and nurses provide clinical oversight, referral, and follow‑up during facility and outreach services, while health officers and physicians support supervision, mentoring, and continuity of care across the health system and in the community.  We have revised the manuscript to more clearly describe these roles. See p6, lines 112-121 |
| In the methods and sampling process, it seems the strata’s could be potentially biased. How was this addressed? | The strata were made between health extension workers and other healthcare professionals, including midwives, nurses, health officers, and physicians. This was deemed appropriate as HEWs are the primary caregivers in the community, supporting, educating and counselling mothers and children, comprising a higher number than other healthcare professionals. To address the sampling distribution, we treated the HEWs as one stratum and the other healthcare professionals (midwives, nurses, healthcare officers, and physicians) as another stratum. See p 7, lines 125 -141 |
| The focus group methodology needs more detail such as who led the session, whether trained in this methodology and example of participants included per session. | We clarify that focus group discussions were not conducted as part of this study. Instead, content validation was conducted through an expert panel review, which may have been interpreted as focus group methodology.  The expert panel consisted of individuals trained in psychometric evaluation and experts in maternal and child health. They did not participate in facilitated group sessions or discussions. Rather, each expert independently reviewed the draft instrument using a structured checklist to rate item relevance and provide qualitative feedback. Content validity indices were calculated based on these individual ratings, and items were revised accordingly. We have revised the Methods section to clearly describe this process and avoid confusion. See p11, lines 221 to 225 |
| Provide the rationale for including which prior KMC surveys were utilized and how the deductive process of not including items were conducted from these surveys. | Items were drawn from prior KMC surveys that were relevant to community‑based settings and aligned with the objectives of this study. A deductive process was used to exclude items related to advanced levels of care, such as neonatal intensive care unit (NICU) practices, which were hospital‑based and not applicable to community settings. See p10, lines 190-205 |
| The ethical considerations need to be moved before data collection, perhaps at end of sample. | The ethical considerations have now moved to before data collection. See p8, lines 144 -155 |
